# Large-scale audience participation in live music using smartphones

**DOI:** 10.1080/09298215.2020.1722181

**Published:** 2020-02-09

**Authors:** Oliver Hödl, Christoph Bartmann, Fares Kayali, Christian Löw, Peter Purgathofer

**Affiliations:** a Faculty of Computer Science, Research Group Cooperative Systems, University of Vienna, Vienna, Austria; b Human Computer Interaction Group, Vienna University of Technology Institute of Visual Computing and Human-Centered Technology, Vienna, Austria; c Centre for Teacher Education, University of Vienna, Vienna, Austria

**Keywords:** Technology-mediated audience participation (TMAP), co-design, smartphone technology, live music, ultrasound communication with high-frequency sound IDs

## Abstract

We present a study and reflection about the role and use of smartphone technology for a large-scale musical performance involving audience participation. We evaluated a full design and development process from initial ideation to a final performance concept. We found that the smartphone became the design tool, the technical device and the musical instrument at the same time. As a technical device that uses ultrasound communication as interaction technique, the smartphone became inspirational for the artist's creative work. In aiming to support the artist, we observed pervasive importance of retaining artistic control to realise artistic intent. This concerns the co-design process and the resulting concept of audience participation and supports recommendations for such participatory work.

## 1. Introduction

Smartphones have become a key device for large-scale audience participation in live music both for scientific studies as well as performing artists, e.g. Hirabayashi and Eshima (2015), Wu, Zhang, Bryan-Kinns, and Barthet (2017), Lee, Willette, Koutra, and Lasecki (2019) or Matuszewski, Schnell, and Bevilacqua (2019).

We contribute to this area of research and build on different promising, but not yet fully studied concepts in the area of large-scale audience participation using smartphones.

In this study, we design, develop, and evaluate Poème Numérique, which is both, a new performance concept and smartphone application for technology-mediated audience participation (TMAP) in live music. We seek deeper insights concerning the role and use of smartphones in live music audience participation. In particular, we want to learn more about the technology itself (e.g. technical issues), the development process (e.g. design decisions) as well as the artistic practice (e.g. composition and performance aspects).

Our methodological approach comprises the design and development process for a participatory performance with smartphones from initial ideation to the final concept. The co-design process with a series of iterative workshops, a large-scale public test and a final performance was conducted involving a music artist. We used interviews and questionnaires to evaluate the experience from both audience and artist perspectives. We found that technology itself became an inspirational instrument for the artist and the artistic practice. This concerns the smartphone in two ways; as a complex technical device itself, and the app and its wide range of interaction techniques that may be used for musical purposes. It furthermore holds recommendations on effective co-design work with artists and smartphones. In the end, we conclude that it is crucial to involve the artist as much as possible in the technical design and development to align mutual artistic and technical possibilities and demands.

We start to give an overview of important work in the area of TMAP and particularly with smartphones. In Section 3, we present the context of Poème Numérique as design exploration and smartphone application. Section 4 explains our methodology and how we combined different qualitative and quantitative methods for data acquisition. This is followed by the results of our knowledge gain in Section 5. Finally, we reflect on the iterative design, development, and test process in Section 6 and discuss the arising implications when using smartphones for TMAP in live music.

## 2. Related work

As discussed in this section, mobile devices, and in particular smartphones are a widely used method for audience participation in live music. This concerns all sorts of musical genres and different interaction techniques to let the audience participate in a concert. Even in the pre-smartphone era, mobile phones were used for participatory performances as we will review first.

### 2.1. The pre-smartphone era

We consider the pre-smartphone era as the time before 2007, when the first iPhone was presented. Two examples for audience participation in the pre-smartphone era are the Broadcast Works by Neuhaus (1994) with wired phones in households all over the United States and Dialtones by Levin (2001) using the ringtones of spectators' mobile phones during a concert. In Neuhaus' performances in the 1960s and 1970s, people could dial a number to become part of a live radio show whereas Dialtones by Levin (2001) has been one of the first examples in the area of mobile phone participation. Before the concert, the audience had to register their phone numbers, take a specific seat in the hall and download a specific ringtone. During the concert, the performers dialled the audience members' mobile phone numbers to play the ringtones via their phones.

McAllister, Alcorn, and Strain (2004) employed Personal Digital Assistant (PDA) devices as part of their participatory concept; spectators used wirelessly connected PDAs to influence the performers. During the concert, the performers could see gestures on screens which were created by the spectators' PDAs. In this report on their study, the authors pointed out the importance of responsiveness and low latency for a positive experience among audience members in the live performance.

Two other examples of audience participation use mobile objects or devices in the pre-smartphone era. In the piece Glimmer by Freeman (2005, September 5–9), the audience uses light sticks tracked by video cameras to interact with an orchestra during the performance. Feldmeier and Paradiso (2007) used giveaway wireless motion sensors for audience participation and reported on positive feedback by the audience using mobile technology for participation.

### 2.2. Related smartphone research foci

The rising popularity of mobile devices, and in particular smartphones, resulted in widening the research foci around audience participation in live music. Oh and Wang (2011, July 31–August 5), for instance, developed and used different audience participation techniques to study social music interaction aided by mobile technology. The technologies they used were custom-built smartphone apps and web applications as well as widely used social media platforms.

massMobile (Weitzner, Freeman, Chen, & Garrett, 2013) was designed to be a flexible and scalable framework for audience participation. In massMobile, the audience uses a web app, therefore participation is not restricted to a specific platform. Each device with an internet connection can be used to access the app via the web browser. Available concepts in massMobile are text entries, voting, sliders, drawings and 2D-sliders. As soon as the user interface (UI) elements appear on the screen, the audience can interact with the given interface. massMobile was tested in various scenarios. In one of these tests, the audience was able to control the lighting configuration with a slider. In addition, a dancer changed his performance according to the light. An interesting outcome was that the audience started to work together to change the light collaboratively. Furthermore, in the study, the authors discuss network latency issues and how it influences use cases in music-related audience participation.

Hödl, Kayali, and Fitzpatrick (2012, September 9–14) observed that distraction can be a critical issue when the audience uses their smartphones for participation in live music. In their studies, the audience was able to control the stereo panorama of the lead guitar by moving their smartphone to the left or to the right. As a feedback loop, the collaborative value of the panorama was visualised by a point on a screen behind the stage. Main findings were that the musicians do not want to lose too much control over their music and that the audience wants to have proof that they exert influence with their actions. People become frustrated if they think that their actions are useless.

A smartphone application where the audience has more control but also plays a different role is called Echobo by Lee and Freeman (2013, May 27–30). In Echobo, the audience uses an app for actively playing an instrument on the phone while a person called master musician is able to change the keys the audience can press on their phones.

Sense of Space by Hirabayashi and Eshima (2015) uses ultrasound as a communication technology for smartphone audience participation. Their smartphone application uses high-frequency sound IDs to trigger sound and visuals on the smartphones and tablets in the audience. These ultrasound IDs are frequencies between 18 and 20 kHz to be recognised by the microphones of the distributed mobile devices. By sending these ultrasound IDs, they triggered sound and visuals on all devices. One problem they encountered during a performance was that the phones' speakers were not loud enough to be perceived in the live situation. As a consequence, the audience members placed their phones near their ears which prevented them from seeing the visualisations on the smartphone screen.

With Open Symphony by Wu et al. (2017) audience members could make creative decisions and generate scores, the musicians were playing in real-time. However, the authors reported that spectators still ‘wished to have more control’ although some others ‘felt challenged by the novelty’. Lee et al. (2019) found out in a recent study that audience members in their crowd performance were ‘actively engaged throughout the performance, with multiple layers of social interaction’ using smartphones as mobile music instruments.

Advances concerning network technology and new software play an important for smartphone-based performances too. Matuszewski et al. (2019), for instance, developed and analysed a JavaScript-based framework for mobile network music systems. With Collective Loops, the authors present an interactive audiovisual installation based on different web standards to allow ‘rapid prototyping, easy deployment and spontaneous participation’ (Schnell et al., 2017).

### 2.3. Popular music and related examples

Smartphone participation has not only been discovered in research but also in popular music. One example is the band Metallica which used smartphone-based voting during their concerts.^1^ The audience was able to vote for three different songs. The voting results are presented directly on a huge screen on stage. A more integrated application was developed by the jazz band Tin Men and The Telephone^2^ or by Dan Deacon.^3^ Both developed and used their own apps to be used by the audience to participate in interactive concerts. The Tin Men and The Telephone app contains real-time voting for soloing and live chatting on a stage screen. The audience in Dan Deacon's app uses their smartphones for light effects, additional sounds and for playing an electronic keyboard.

A simple smartphone-based audience participation was used by Robbie Williams.^4^ During a concert in a stadium, he asked the audience to use the flashlight of their cameras at the same time which led to a massive light effect.

In comparison to most smartphone interactions in this regard, the app ‘Don't miss the buffet’ from The Salome Experience by Reichl et al. (2016) does not focus on the performance itself but rather on the information an audience member misses when he leaves the performance room for some minutes.

The interactive piece Experience by Hödl, Fitzpatrick, and Holland (2014, September 14–20) does not use smartphones at all, but studies audience participation in a popular music context. In particular, the authors study the composition of a rock song for audience participation and its later performance.

## 3. Design exploration poème numérique

Poème Numérique denominates a design exploration for large-scale audience participation in live music. Poème Numérique stands for a smartphone app as well as an interactive performance with the same name where audience members use the Poème Numérique app to participate. This was inspired by similar approaches using smartphones for large-scale audience participation such as massMobile (Weitzner et al., 2013) and Echobo (Lee & Freeman, 2013, May 27–30). Furthermore, we built on the technical concept to use ultrasound communication as in the Sense of Space (Hirabayashi and Eshima, 2015).

This work arose from a research project and performance series called Breaking The Wall.^5^ The project Breaking The Wall,^6^ however, gives the broader context only at this point and is summarised in Kayali et al. (2017). With Poème Numérique, we focus on one particular interactive performance or rather its whole preceding design and development process in two phases as described next.

The performer, Susanne Kirchmayr, as an artist known as *Electic Indigo*,^7^ is an Austrian composer and musician. Surround sound as well as temporal placements of elaborate electronic sounds are important elements in her award-winning music.

The Poème Numérique design exploration was done in two phases. The goal of the first phase was prototyping the smartphone app Poème Numérique. This included the design and development in iterative workshops and a large-scale feasibility test.

The second phase covers the improvement and performance rehearsals of Poème Numérique. This phase encompasses several workshops at the actual performance venue and the final public performance. We refer to Kayali et al. (2018) and Kayali et al. (2017) for detailed documentation of the workshop process developing the Poème Numérique app.

## 4. Methodology

Exploring the design space of audience participation with special regard to smartphones as interactional modality implies an interplay of perspectives: concert-goers and users of smartphones bring to the table a variety of expectations, experiences, and ideas around using their smartphones in general and during concerts in particular. Artists might consider the audience participation via smartphone as part of an artistic performance tool set or musical instruments and express interest in augmenting, evolving or extending their artistic practice.

In the design exploration of Poème Numérique, we as researchers and designers of technology assumed an intermediary role in relation to users (inquiring and explicating expectations and needs) as well as the artist (facilitating artistic involvement with technology design) and conducted an exploratory design process along several methodological steps:

1. *Initial Ideation.* Semi-structured interviews were conducted with potential audience members and musicians (see Section 4.1).
2. *Participatory Workshops.* Together, four experts and the artist designed and implemented an audience participation application for smartphones as a functioning prototype and improved it along two iterative phases (see Section 4.2).
3. *Evaluation.* Integrated in and accompanying the workshops, the concept and the prototype was evaluated via questionnaires in a large-scale live test with a test audience. Two semi-structured interviews, one after the live test and one after the actual performance were conducted with the artist to capture experiences, reflections, and needs (see Sections 4.3 and 4.4, respectively).

**Figure 1.**
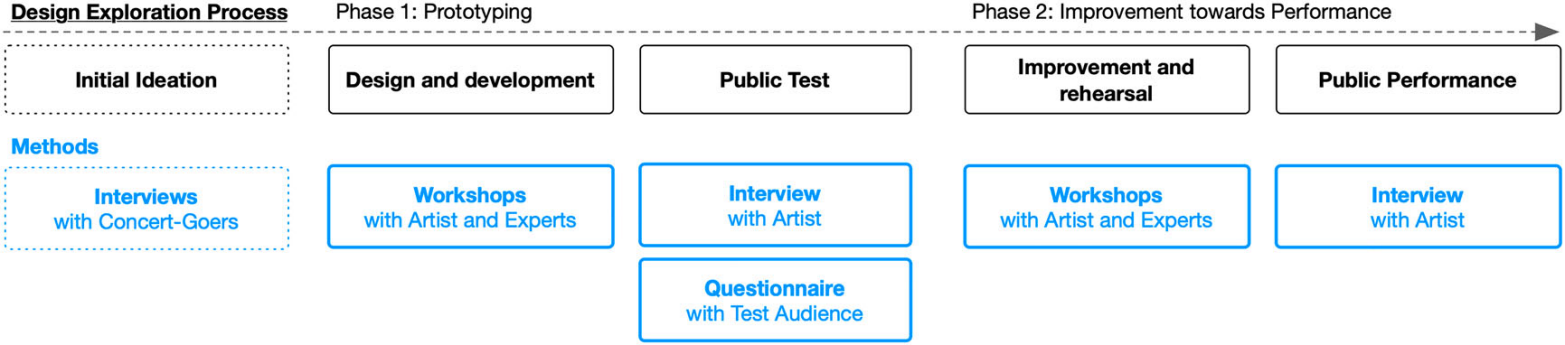
Methodology overview.

Figure 1 gives an overview of the process and overall outcomes. In the following sections, the employed methods are described in detail.

### 4.1. Semi-Structured interviews with concert goers and musicians

Our goal in the first place was to find new possibilities to use a smartphone for participation during a concert and to get a basic feeling of what concepts people expect, want or even reject. Therefore, we chose semi-structured interviews with audience members as well as musicians who are smartphone users. We conducted interviews with four people who had the following characteristics: The person is a smartphone user, likes music and goes to concerts. The person went to concerts in the last months. In addition, we interviewed three musicians who are smartphone users as well.

During all interviews, we asked general questions about concerts, audience participation and interaction between the performer and the audience. Furthermore, we asked questions about smartphone participation methods and tried to brainstorm about possible new methods with all kinds of sensors a smartphone has.

### 4.2. Design workshops and iterative development

After ascertaining what audience members as well as musicians expect from an audience participation application, an application was iteratively developed and reviewed in several sessions set up as design workshops. The participants comprised an artist/performer and four other experts coming from arts, music, sound engineering, game design and computer science. We chose the focus group because an interdisciplinary topic may be solved better by an interdisciplinary team (Kitzinger, 1994). The input we used were the ideas which we raised from the preliminary interviews.

### 4.3. Questionnaires with a test audience

In the course of the design process, the smartphone application prototype was evaluated formatively in a large-scale live test. During the event, a questionnaire was handed out. Additionally, the focus group and the performer acted as moderators as well as observers.

The questionnaires contained eight single choice questions and six open questions. For one single choice question, there was an additional single choice question if the answer was *Yes*. Two of the open questions only had to be answered if the corresponding single choice question was answered with *Yes*. If answers were missing, we still classified the questionnaire as valid. The reason for this is that it is possible to evaluate the single choice questions independently from each other. For the open questions, no answer is also valid. The only questionnaires we counted as invalid were questionnaires where half of the questions or more were unanswered and where we had the impression that the person did not understand the questions asked. We indicate that the amount of people who answered the open question is not the same amount of valid answers per question. The reason is that some people wrote more than one answer, some people wrote useless or incomprehensible answers and some answers were categorised in more than one category.

Roughly, 250 people participated in the live test, and 221 filled-out questionnaires were obtained. For analysing the open questions, we used thematic analysis (Adams, Lunt, and Cairns, 2008; Braun and Clarke, 2006).

### 4.4. Semi-Structured interviews with the artist

To obtain an in-depth understanding of the artist's perspective on top of her participation in the design workshops, two semi-structured interviews were conducted at the end of phases I and II of the design process.

The interviews were conducted at the end of phases I and II after the public test and the public performance, respectively. The questionnaire covered her experiences with using the prototype, her impression of her audience during the performances, the process of appropriation and integration of the provided technological means into her artistic practice and an exploration of further development. The interviews took around 30 min each and were audio-recorded and subsequently transcribed for analysis.

## 5. Results

In this section, we present the results of the three methods we used, starting with the results of the preliminary interviews, followed by the results of the workshops and the evaluation of the questionnaires. The outcome of the previous method stands for itself and was used as input for the consecutive method (see Figure 1).

Depending on the respective method and nature of obtained data, different approaches to analysis were taken: Open questions were analysed towards the formation of themes by means of thematic analysis. This applied to the semi-structured interviews on ideation (results given in Section 5.1) and with the artist (results given in Section 5.5) as well as to the questionnaire (results given in Section 5.3.2). Descriptions of the workshop process (results given in Sections 5.2 and 5.4) ?>constitute documentation and reflections by the researches personally involved in the workshop process. Closed questions of the questionnaire were analysed and interpreted statistically, and results are given in Section 5.3.1.

### 5.1. Initial ideation

The participants mentioned various possible use cases based on the different technologies typically integrated in a smartphone. The following list groups the results by smartphone functionality; given this method's goal of open ideation, it represents a collation of ideas, stemming from the participants' experience with attending musical concerts, sometimes accompanied with anticipated or experienced drawbacks.

The *Integrated microphone* could be used for singing, or letting one randomly chosen person sing. *Integrated speakers* could be used for playing sounds collectively or for synchronising collective actions like jumping. However, speakers of the phones could possibly not be loud enough to realise such ways of mass interaction. *Localization* features like GPS could be used for rating the sound, the light show and/or stage experience by interpreting proximity. The location could help to identifying ‘good’ or ‘bad’ place in the audience. Localization features could furthermore be used for recording the movement of the audience; audience members could watch the movement on their screens. The *display* could be used for live streams with individual camera selection. It could be used for displaying various forms of live second-screen information like lyrics, a schedule, current title and album, the total and elapsed length of a concert, volume or further links to music stores. It could also be used for creating a light show similar to a sea of cigarette lighters or Xylobands.^8^ Participants could furthermore request and auction for a specific light show, which is then conducted by all involved smartphones. *Integrated cameras* could be used for sharing pictures and videos on a platform. Instead of prohibiting the audience from taking photos during the performance, the band could profit from free media exposure and the audience could profit because they can download all the media created from other concert visitors, a feature which was however also suspected to be a possible distraction from the concert experience. Lastly, *integrated actuators* could be used as a metronome for the musicians and/or the audience or as an additional multimodal effect augmenting the light show. Furthermore, it could be used for synchronising interactions like jumping.

As general drawbacks mentioned during the interviews, it was mentioned that mobile reception might not be satisfactory at concerts, and that it could be distracting for musicians to actively take part in an audience participation concept with smartphones.

These results were provided as inspiring inputs, as something to possibly work with for the artist and experts in the subsequent workshop phases.

### 5.2. Phase I: Design and development workshops

Across the two design process phases, in this first phase, workshop sessions were held over the time of ten months. Along this paper's focus on smartphones as a technology for this audience participation design project, the following paragraphs give more regard to technical design considerations and decisions made in the course of the workshop. Methodological description of the workshop process can be found in an adjacent original publication on this project (Kayali et al., 2018, chapter 3). The development process towards the final Poème Numérique application comprised four steps:

1. *Select a technology for the smartphone app.* It was most important for us to allow participation to as many people as possible. As we also wanted to have most and direct access to native smartphone functions and sensors we went for Xamarin,^9^ which is a cross-platform framework for app development, instead of using a web app. With Xamarin, we were able to develop a single application for iOS, Android and Windows Phone. For prototyping, we only developed for iOS and Android, as these two systems are the leading operating systems on the smartphone market. Cross-platform compatibility at the same time limited what we could do with audio files, such as using filters or more precisely controlling playback.
2. *Select a smartphone interaction technique.* We reviewed and considered different ways of wireless interaction techniques with smartphones. Our decision was to follow an approach by Hirabayashi Eshima (2015) and develop an application which reacts to high-frequency sound IDs. The reason for that was that triggering events with high-frequency sound IDs is faster than through 3G, 4G or Wi-Fi, not dependent on cell reception or signal power and there is no need for network infrastructure. Moreover, reducing wireless network traffic or even activating flight mode at the same time reduces the battery drain.
3. *Implement a first app prototype to test chosen approach.* This first app prototype was able to play local sounds stored within the app triggered by ultrasonic sound. During these tests, we also found different colours as full-screen display backgrounds to be an interesting augmentation of the sounds played locally on the phone. We found that randomness adds to the experience, meaning that one trigger sent by the artist can trigger one of a number of sounds. During this step, the artist also got a feeling of how to compose for this app by taking advantage of overlapping, repeating and spatially distributed sounds played by different smartphones at the same time.
4. *Optimise app.* Finally, we optimised the app concerning the ultrasound technology and its stability. At the same time, we identified technical restrictions of the recognition of high-frequency sounds such as the rooms' shape, the frequency volume and the quality of the microphones of different smartphones models.
5. In theory, we are able to use more than 50 high-frequency sound IDs (Bartmann, 2016). Unfortunately, the reliability of the recognition drops when increasing the number of IDs. What we observed during the development is that the recognition of lower frequencies works better than of higher frequencies.

**Figure 2.**
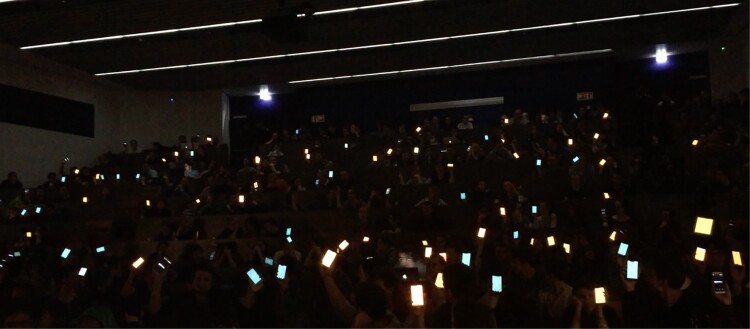
The audience holding their colourful displays up in the air during the large-scale test. They were asked to hold up their devices to see the effect.

**Figure 3.**
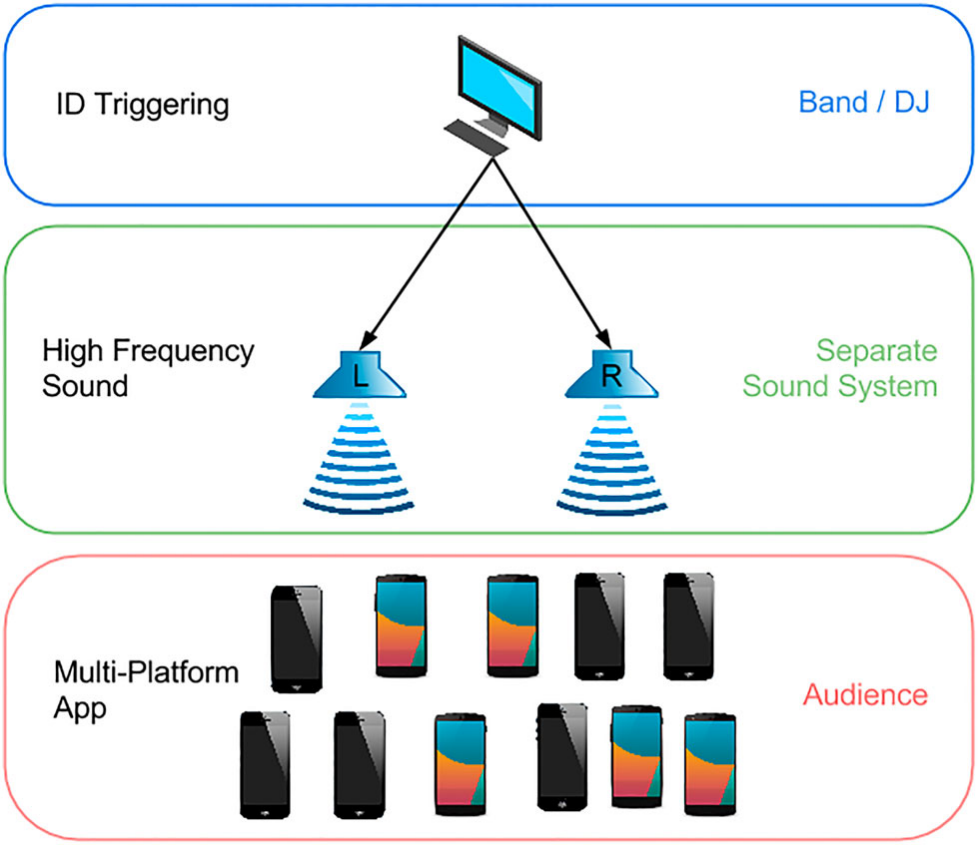
Schematic diagram of the technology behind Poème Numérique taken from Kayali et al. (2017).

The phase I workshops resulted in the first iteration of Poème Numérique, a smartphone application capable of reacting to ultrasound cues triggered by the performance artist by (1) playing pre-recorded sound samples through smartphone speakers and (2) contributing to a collective display light show (see Figure 2). The Poème Numérique app is available for iOS and Android. The app reacts to specific high-frequency sound IDs by playing pre-recorded sound files and showing blinking full-screen colours on the audiences' smartphones. The performer is able to trigger 15 unique IDs between 18 kHz and 20.7 kHz. Each ID triggers a specific combination of sound and visuals at all participating smartphones. Note that the number of participating smartphones in the audience is not limited. Only the number of IDs to trigger sound and visuals within the app is limited to 15. See Figure 3 for a schematic diagram of the technology behind Poème Numérique.

### 5.3. Phase I: Public test and questionnaires

**Figure 4.**
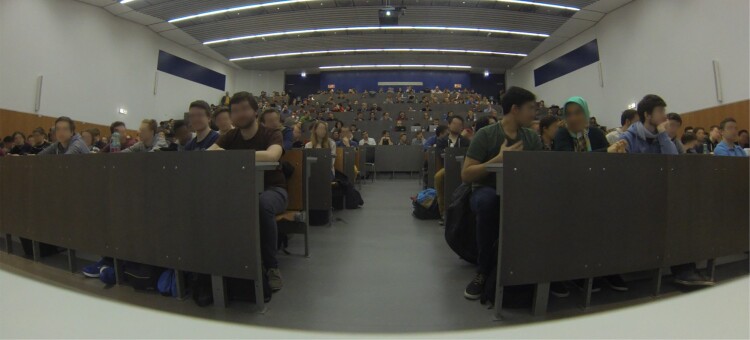
The Audimax during the large-scale test photographed from the front table.

Following prototype creation, a public test was carried out to evaluate the reliability of the utilised ultrasound queuing mechanism and gather experiences regarding the overall artistic effect. The test took place in a university lecture held in the largest lecture hall of Vienna University of Technology, was attended by undergraduate computer science students (see Figure 4) and consisted of a brief introduction, short musical performance by Electric Indigo and the hand-out of a post-test questionnaire.

221 people filled out the questionnaires of which two questionnaires were counted as invalid. 18.7% of the participants were female, whereas 1.8% did not declare their sex. 85.4% of the participants were between 18 and 25 years old. 2.7% did not declare their age. 11.9% were between 26 and 40 years.

#### 5.3.1. Single choice questions

Overall, the evaluated prototype was well-received by the test audience: 72.1% of the participants did install and use the app on their own smartphone during or before the live test. 92.7% liked the concept of the application, 67.6% can imagine using the app in a concert and 71.7% think that the app can improve the concert experience.

Anticipated possible criticisms were present, but not predominantly so: 34.7% of the participants thought that the app is distracting during concerts. 46.1% thought that it is annoying that they cannot use their smartphone for other activities (like photos, filming, communication,…) during the concert. 37.0% wished for (more) possibilities for interacting with the application during the live test. Although 39.3% of the participants were able to hear the high-frequency tones only 17.8% found them distracting.

#### 5.3.2. Open questions

The open questions of the questionnaire were put in place to explore further opinions expressed in closed questions and get more detail on general views regarding the prototype.

*Views on distracting qualities*. 79 people answered the question ‘Why do you think that the app is distracting during concerts?’ which was only explicitly posed to participants who thought that the app would be distracting during concerts.

The most frequent answer was that the use of the app itself is *distracting in general* (21) without going into detail, some more participants specified it further as keeping the smartphone in their hands for prolonged periods being tedious (8), others attributed a feeling of distraction to smartphone usage during concerts in general (8). Another commonly mentioned source of distraction was *sound quality*. Several participants did either not like the sounds it produced in general or thought they did not fit well with the music on stage (11), some others mentioned that the sounds were asynchronous (9). A general reservation beyond the live test at hand expressed by some participants was that a distracting quality might result from a *bad fit to the musical performance*, with possible distractions depending on concert type and genre (9), how it is integrated into the performance (6) or clarity of explanation to the audience (6).

*Views on the relation to the concert experience*. 146 people answered the question ‘Why do you think that the app can (not) improve the experience of a concert?’. Apart from several ambiguous replies by participants who felt that the purpose of the app was not clear (16), that it would depend on the concert it is used in (10) or on how well its activity is synchronised to the concert (4), several positive and negative replies were obtained:

The most frequent *positive answers* were that the app generates group feeling, the feeling that everyone is part of the concert or that the app promotes audience participation and audience interaction (33). Others mentioned that the app is new and interesting (20) or that the app improves the acoustics, the sound-effects or the surround feeling (18). The most frequent *negative answers* were that either the visuals, the sound or the app are annoying (9), that smartphones are not loud enough (7) and that one's focus should be centred on the stage and the music of the performer (6).

*Views on the overall experience*. 181 people answered the question ‘What did you like about the app?’ and described *positive experiences*: By far, the most common answer was that the person liked the idea or the concept of the application (75). The next most common answers were that the person liked the colours or the light-show (27) and that the person liked the technology used within the application (25). Some mentioned they liked that the application or the handling of the app was simple (16), they liked either the group-feeling or the interaction by using the app (14) and they liked the surround sound or surround light feeling (13).

158 participants answered the question ‘Did you have problems with the app? If yes, what?’. 79 affirmed this and described *negative experiences*.

Some people answered that their phone never reacted on the IDs (37) or that their phone was not reacting at the beginning (14). Some also mentioned that their phone only reacted sometimes (12). In some cases, it was not clear whether the person had a problem (8). Others mentioned that there was a problem but did not specify it (6). Less frequent answers were that the app crashed (3) or that the app only reacted when the person was holding their phone in the air (3). Considering smartphone operating systems, it is feasible to assume that there is a problem with operating systems which are not directly supported by Xamarin likes CyanogenMod or Oxygen OS (nine out of 14 users of CyanogenMod reported they had a problem with the app during the test).

*Views on additional forms of interaction*. The question ‘Which additional interactions would you wish for?’ was posed to participants who expressed that they wished for more interactions and answered by 64 people. The most frequent answer was that the person wanted to choose the displayed visuals (19), followed by choosing the sounds (17). A few people also would like to produce the sound in some way (5) or vote for something (4). Others wanted to have some kind of input possibility, a menu or options (4). Also, media support (filming, photos, messaging), feedback for the performer, additional information, shake functionality and wave-like spreading of sounds or visuals were among the answers.

*Other suggestions for improvements*. 164 people answered the question ‘What could be improved in the app?’: People answered that the app should be compatible with more devices or that the application should be more stable (16). Others mentioned that more interactions should be added or that interactions should be improved (15). Participants also wrote that more information should be provided (14), that other operating systems should be supported (13) and that the colours should be selectable or more colours and multicoloured screens should be provided (13). Some (10) mentioned that more functionality should be added or that the application should be able to run in the background to enable other functionality (10). Less frequent answers were that the sounds should be changed or improved (8), that health should be considered (i.e. epilepsy warning) (8), that the user interface should be improved (8), that the download size of the application was too big (7) and that the sounds or lights should be more synchronised (6).

#### 5.3.3. Summary of the public test

In general, the concept of Poème Numérique was highly accepted in our specific test. However, we observed issues with the recognition of high-frequency sound IDs with specific smartphones and the general low volume of the sounds played by the app. The location of the spectator in the room as well as the room shape influences the recognition quality and the volume of the smartphone-generated sound. In case of the volume, the sound generated by all smartphones together was much lower than we thought. Like with Echobo (Lee and Freeman, 2013, May 27–30), in our case some people also complained about the limited volume. With the room shape and distance to the sound ID sending speakers, we found out that the lecture hall we used during the live test uses multiple methods for noise reduction from the audience area. Reduction methods include specific wall panels, holes in the ceiling, adjustments in the relative height of the seat rows as well as the use of a specific flooring. Concerning the ultrasound itself, some people in fact could hear the high-frequency sound IDs, but it did not bother them much.

### 5.4. Phase II: Improvement towards performance

After the large-scale live test the improvement and rehearsal phase started. In six workshops over a year, the Poème Numérique app was continuously improved along with the artistic and conceptual development of the performance. In two of the six workshops, the artist was not attending as these two were focusing on crafting and technical issues only. These workshops and their goals were:

1. *First trials to amplify the sound of single smartphones with pickup coils.* This was a design decision based on the outcome of the public test where the sound of the distributed smartphones was too silent.
2. *Further improvement of the pickup coil idea and testing the smartphone screen blinking.* These further tests with pickup coils were already done with four distributed speakers similar to the final setup. The improved Poème Numérique app was tested with new sounds and blinking colours according to the artist's concept.
3. *First prototypes of the stations with polystyrene cubes and 3D printed smartphone mounts.* This was the only workshop without the artist as the focus was crafting the stations.
4. *Testing new sounds with the app and the final station concept.* The Poème Numérique app was tested with new sounds according to the artist's concept. This test happened with four stations like in the final setup, but only one station was made of wood according to the final station design.
5. *Finalising the four stations for the performance.* In this workshop, the four final stations were built. Again, the artist was not attending as the focus was woodwork, painting and wiring each station as well as 3D printing and laser cutting the smartphone mounts.
6. *Final setup and general rehearsal on the day before the performance.* During the rehearsal, the artist decided that the sound of the pickup coils amplifying single smartphones was too disturbing. Apart from this decision, the final setup and concept of the stations appeared as described in the following.

**Figure 5.**
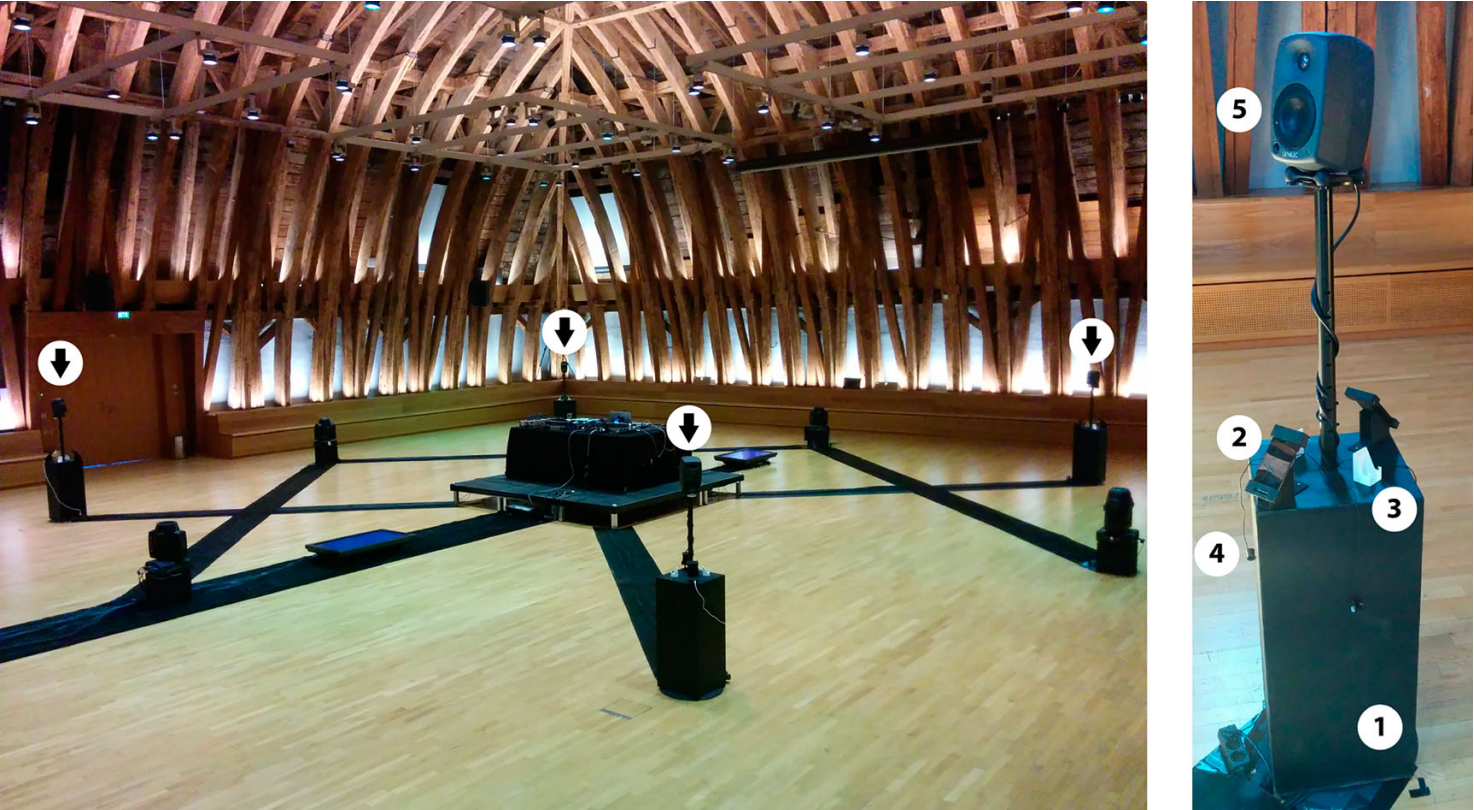
Left: The performance venue with the centre stage for the DJ and four marked stations. Right: A close up of one station with (1) its base, (2) mounted smartphones, (3) empty mounts, (4) pickup coils, and (5) a speaker as further described in the text.

We see the final performance setup at the venue in Figure 5. The rehearsals during the year before the performance took place at the same venue with a similar setup, however, without an actual stage and the final four stations as in Figure 5, left.

**Figure 6.**
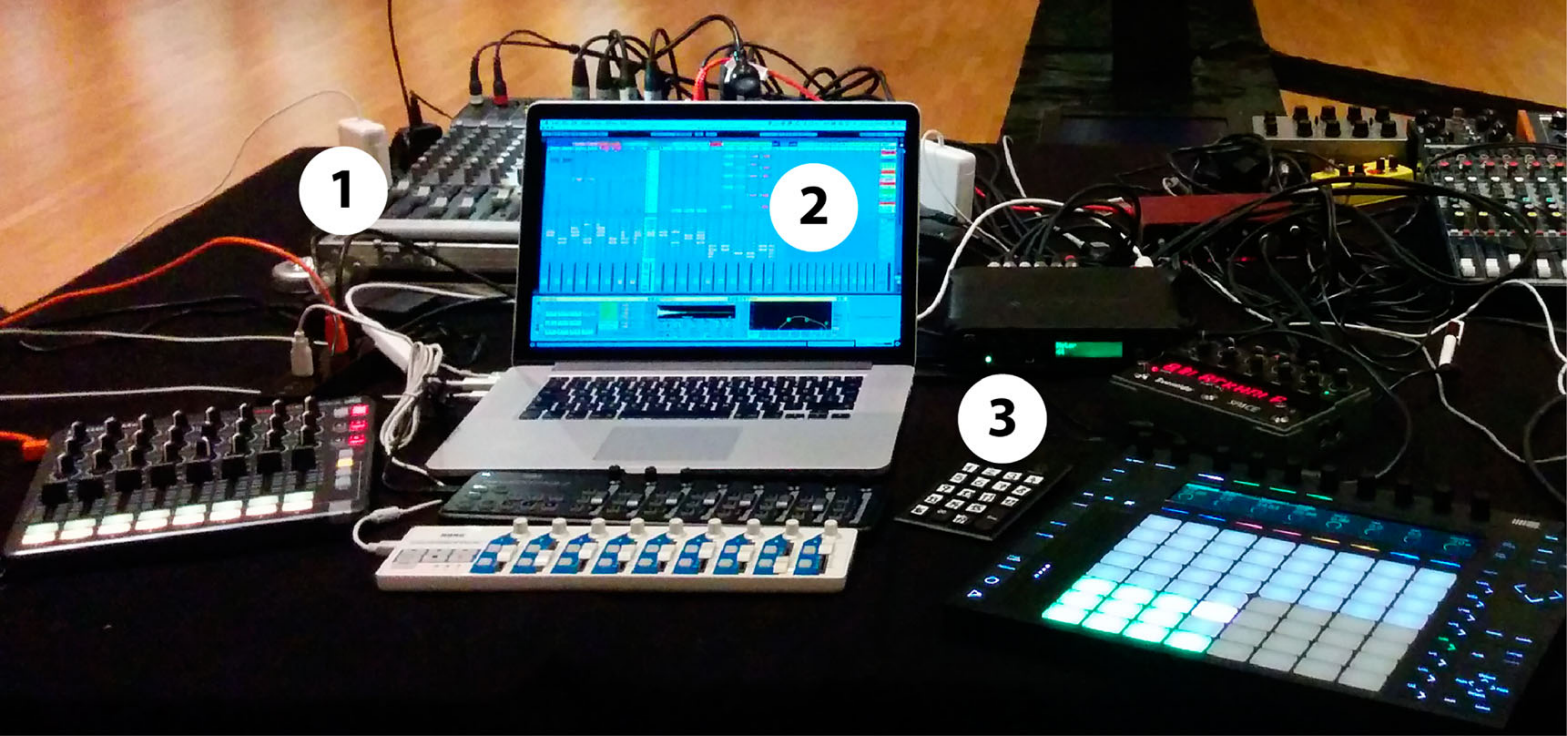
The DJ setup at the centre stage with a (1) mixer, (2) laptop, and (3) keypad to send ultrasound IDs as further described in the text.

*The centre stage* contains the DJ setup as shown in Figure 6. With the mixer (1) the DJ has full control of the sound in the hall and can use six speakers: two stereo PA speaker and four speakers on the stations around the centre stage. The laptop (2) is the main instrument of the DJ with additional control interfaces placed around the device on the table. With the keypad (3) the DJ sends the ultrasound IDs that controls the Poème Numérique app on the participants' smartphones.

*The four stations* are all alike as in Figure 5, right. Each station has a hollow wooden base (1). On top of this base are four smartphone mounts. There are two black wooden laser cut mounts with actual smartphones (2) for spectators who do not have a device or do not want to use their own device. The other two empty mounts are white 3D printed (3) and intended to invite participants to temporarily mount their smartphone while using the pickup coils. Each station has four of these pickup coils loosely wired attached (4). These pickup coils are intended to be used by spectators to amplify electromagnetic signals of the smartphones. The pickup coils are not directly amplified at the station but sent to the DJ mixer on stage. The speakers of the station (5) are controlled centrally by the DJ mixer as well and form four additional locally distributed speakers to the PA's stereo speakers.

Concerning the final acoustic setup, the DJ has full control of the sound sent from all four stations to the stage by participants using the pickup coils and how the sound is played through a ‘6.1 speaker system’ in the hall. The PA setting can be considered a 6.1 surround system somehow as there are two stereo main speakers, the four distributed station speakers and one subwoofer.

### 5.5. Interviews with artist

In the course of the Poème Numérique Design Exploration, two semi-structured interviews were conducted with the involved artist, Electric Indigo^10^ (see Section 4.4 for methodological remarks) to capture her artistic perspective on the process.

A meaningful theme that emerged from what Electric Indigo considered important for her work is artistic control, which manifested itself three-fold: (1) Being *in control of the means* – which the artist described as being like the mastery of any other musical instrument, (2) creating a tool that enables the artist to be *in control of the sound*, which implies a tool that is capable of creating well predictable, finely controllable/modifiable sound and (3) being *in control of the performance*: While well-defined degrees of freedom exist that are assigned to the audience as spaces of participation, the overall architecture of the musical composition should remain under the control of artistic intent. This, together with overall impressions of the Poème Numérique design exploration, is exemplified in the remainder of this section by remarks made by the artist on the design process, prototype development, performance experience and possible future steps.

#### 5.5.1. Co-creation and creative practice

The design exploration was undertaken in this project aimed at embedding creative practice in a setting of technological co-creation. To the artist, the resulting way towards Poème Numérique took shape as an iterative excursion from known towards more unknown fields of curiosity and artistic interest: ‘Because I could not imagine lots of smartphones as an instrument, we did these tests and I just tried out sounds of various kinds. [..] I took a piece, of which I knew by experience that it is well suited for experimentation, which offers enough sound space to add other elements and which is furthermore structurally open enough to experiment with timing and sounds’.

The artistic process described by Electric Indigo is led by their her core interests - composition and performance: ‘If I would use [a technological prototype], I would compose a piece for it, just as, for example, I compose a piece for eight speakers and that is a part of all considerations from the beginning’. This implies a need for artistic control of the performance and its technical parts, from a conceptual point of view: ‘If it is a solo concert, the theoretical idea behind a piece is important to me. It actually gives me further ideas for concrete aesthetical content. Structure and progression of composition is enormously important to me’.

Exerting control over the performance emerged as a core interest not only in respect to involved technology and resulting composition, but also to the performance situation itself: ‘I have projects, where timing is extremely important and the relation of [sound] elements to one another is totally defined – conceptually, there is no space for temporal fluctuations. But in most projects, I decide by instinct [in the moment], how long different passages would last, what is added and what is not’. Artistic intent, in this sense, thus requires control over the performance both beforehand and in the live situation.

#### 5.5.2. Prototype development

Electric Indigo described that her first idea was to create granular clouds. Because this turned out to be unrealistic, she realised another idea: ‘Using smartphone speakers as additional speakers for a multichannel concert interested me most’. The workshop team was surprised by the fact that 25 smartphones in a performance situation would create a clearer, more concise sound than what similar tests with 200 smartphones would result in. From an artistic point of view, a blurred soundscape is unsatisfactory to the artist, which led her to formulate further goals in mitigating this: ‘There are two ways in which I would like to conduct further tests. One is making [smartphone sounds] even more sparse, which leads to silent periods, but at the same time might reduce this blurring effect a little. And the opposite direction, meaning that I do not want want to reproduce specific musical structures with it, but rather Layers and Drones’. Being in control of the sound emerged as an important goal to the artist, surpassing specific artistic ideas: ‘One just has to work with these specifics and not try to work against.. to do something against the nature of the smartphones which then does not work well’. Exploring the ‘nature of smartphones’ has to be supported by the participatory design process.

For the artist, her intent at times was hard to reconcile with the audience participation: ‘It always is a balancing consideration between artistic aspiration and the aspiration, to what extent the audience can or is allowed to participate’. Within these sometimes conflicting roles, incorporating interactivity and supporting audience participation, however, was an aspiration for her: ‘People would have had more fun with [Poème Numérique’] if they were able to interact more'. In the course of the process, the inherent conflict between artistic intent or being in control of the performance on the one hand and giving the audience degrees of participatory freedom on the other was decided in favour of the former: ‘By not using the pick up stations, I decided in favor of the musically more interesting result and in doubt put the possibilities for interaction aside’.

#### 5.5.3. Performance experience

Electric Indigo had a positive impression of the audience and characterized them as open and curious, as well as a good mix in age. While generally content with the performance, she described concerns about possible confusion and thus distraction among the audience in regard to Poème Numérique, limiting possibilities of audience participation: ‘For the interaction part to work better, people would have to have more information beforehand, they need to be told, there are triggers that fire sounds on the smartphone’.

Another learning of the performance was directed at sample length: ‘We had experimented [beforehand] what happens when short files are looped more often and there is off-set [in starting time] and whether this is interesting soundwise or not, and it turned out that is not particularly interesting, because they start off with individually different latencies anyway [..]. When testing [beforehand] a minute seemed terribly long to us, but in a half-hour concert it is not that long.’. In this, sufficient control of sound had not been achievable, thus artistic intent changed form.

Lastly, the application, from her point-of-view, exhibits poor usability and is *intransparent*.

#### 5.5.4. Conclusion and future steps

Overall, the artist was happy and satisfied with the process and outcome (‘There is potential in [Poème Numérique].’) and expressed interest in continuing it, to either explore new ideas or refine and further existing ones:

An idea that resulted from the workshops mentioned by the artist was to further extend the artistic control over sound and performance and allow for more fine-grained and immediate changes: ‘All possible controls would be interesting [..] volume control, control over what is played where. If [the resulting sound during a performance] is uninteresting, not having to wait until the end of the sound, but rather that I'm able to say ‘stop’. Maybe an equalizer, too. [..] That's something I'm itching to do.. if I hear ‘ok, there are too many mid-level sounds’, then I'd like to be able to reduce them while it's running’.

Include more time and opportunities for artistic practice to further the control of the means: ‘I think, it is somehow like a practice thing for me, controlling [Poème Numérique] well. I would explain people beforehand what it is and what they can do with it’. ‘I would like to practice more. This is the same as in every artistic activity. [..] It is like an instrument, that wants to be played. [..] It gets really interesting, once one becomes fluent with a material and the possibilities’. Electric Indigo suggested having a number of performances as more live situations would be beneficial for testing out possibilities *in situ*.

Lastly, the artist mentioned she was interested in revisiting audience participation and searching for a way to better integrate it into artistic intent: ‘I would make it a priority to find out from the very beginning, how the interaction could be included. [..] I don't yet know which forms of musical interactions could also bring a positive musical result. Currently, they are quite passive. What would happen if they could interact musically and more actively?’

## 6. Discussion

The whole process around Poème Numérique allows us to reflect on implications when using smartphones as design material in large-scale TMAP applications. For this reflection, we take the role of the technology itself into consideration, our experience during the iterative development and testing process and the artist's perspective.

### 6.1. Technology-driven factors

We start to reflect on our smartphone app Poème Numérique and the technological implications when using a smartphone for large-scale technology-mediated audience participation. We rely mainly on the experience from the iterative development workshops during phase I and II as well as the public test.

In contrast to Sense of Space by Hirabayashi and Eshima (2015), which inspired us to use ultrasound communication, we used a cross-platform development environment and used a different evaluation method in a larger scale. In particular, we offered two apps for both iOS and Android and evaluated the spectator experience using questionnaires. We can assert that using a cross-platform development environment enables to reach as much of the audience as possible. At the same time, it has to be considered that cross-platform development imposes restrictions regarding software features.

Using a cross-platform app platform voided the use of platform-specific audio processing which would have enabled more flexibility and lower latency. We also recognised custom operating system mods (e.g. CyanogenMod) as problematic for running our app. The prevalence of these mods definitely was much higher than usual in our specific setting (computer science students), but their existence still has to be considered regarding software compatibility and app platform choice.

The issues we observed with unreliable ultrasound recognition and the low volume of smartphone speakers to play sounds by the audience were also reported by Hirabayashi and Eshima (2015). The use of smartphones as additional distributed speakers or rather the low sound level has to be considered carefully (see also Lee and Freeman (2013, May 27–30) and their study with Echobo). Another observed issue of smartphone sound to be considered when utilising smartphones in (mass) settings of audience participation is a blurring effect probably caused by poor device coordination, as described in Section 5.5.2.

### 6.2. Experience-driven factors

We proceed with reflecting on the implications by the experience when using smartphones for large-scale audience participation. We rely mainly on the experience and opinion of the participants at the public test and the iterative development phases before and after the public test.

In Poème Numérique, a smartphone and to download and run an app are necessary to join the participatory performance. This has a series on implications for the experience of audience members.

Usually, in a phone-based interactive performance, the participants need to have the right device and operate it properly to participate (Hödl et al., 2012, September 9–14; Lee and Freeman, 2013, May 27–30; McAllister et al., 2004). The live radio shows of Neuhaus (1994) and the cellphone performance Dialtones Levin (2001) make an exception as attending or joining the performance automatically means that people are involved. Although a device is still necessary to participate.

With Poème Numérique, we offer different strategies to enable audience members a participating experience even without having a device and without using it a lot during the performance. First, with the four stations and mounted smartphones, people could participate without having a device or without using their own. Second, we focused on an unobtrusive interaction concept that uses ultrasound communication, which even works in flight mode, and an app which does not expect user interaction at all. The potential distraction of smartphones in participatory performance was a general issue by the people we asked during the initial ideation interviews, the participants of public test and in literature (Hödl et al., 2012, September 9–14).

The public test participants also remarked the need for clear instructions of how to use the app. This concerns general information beforehand to let the audience know that they will need their smartphone during the performance. Ideally, that have downloaded the needed app already when the concert starts and have their phones fully charged. In our particular case, the Poème Numérique app did not allow user interaction which could have been communicated. This was intended to prevent distraction, but the test participants thought they could not use the app properly due to missing user instructions.

### 6.3. Artist-driven factors

Finally, we reflect on the implications from an artistic perspective. These implications for a smartphone-based participatory performance are mainly driven by the interviews with the artist, Electric Indigo, who was involved from the beginning.

From the point of view of Electric Indigo, the design process of Poème Numérique placed her in the role of an expert, who, together with the other workshop-participants, would co-create prototypes, try them out and reintroduce these artistic experiences to the workshop-phases in an iterative manner. This unfolded into an iterative process that supported Electric Indigo in an artistic excursion and, in subsequent analysis, yielded a view on her perspective and needs as an artist working with smartphones in her art. Acquiring and maintaining *artistic control* in a project as a prerequisite to realising artistic intent emerged as a dominant theme from the artist's reflections on the design exploration process as described in Section 5.5, which in turn holds implications for Co-Design processes that are aimed at supporting performance artists:

1. Processes aimed at creating musical instruments need to provide performance artists sufficient opportunity to acquire *control of the means*, as artistic work with musical instruments requires time and opportunity to practice.
2. To realize artistic intent, resulting musical instrument prototypes need to exhibit sufficiently fine-grained *controllability in respect to the sound they produce*, both generally and in the live situation.
3. Based on the remarks of Electric Indigo, it is feasible to assume that artistic intent also encompasses audience participation as need for being in *control of the performance*. Concepts involving audience interaction and involvement in sound creation in situ should shape such ways of participation not as something taking place separately from or in addition to a musical performance, but as extension of artistic expression, and provide technologically supported ways of participating and related degrees of audience freedom to the artist as a part of their ‘artistical design space’.
  The related field of tension between artistic control on the one hand and the inherent value of technologically supported audience participation as a means of inclusion and democratisation should be reflected on in such participatory work and, if possible, be made accessible to and usable by artistic intent.

As reported in other studies (Hödl et al., 2012, September 9–14; Lee and Freeman, 2013, May 27–30), keeping control in a performance with audience participation is an important issue for artists. Musicians on the one hand want the application to improve the experience for the audience and on the other hand they also may not want to lose too much control over the music during their concerts. In Echobo (Lee and Freeman, 2013, May 27–30), the ‘master musician’ has full control over the harmony and the audience can only play notes within the given harmonic scale. Hödl et al. (2012, September 9–14) also figured out that ‘musicians seem to be cautious about giving up control’ in their audience participation concept. They let the audience collaboratively control the stereo sound with their smartphones. Similarly to both of these two approaches, the performer in Poème Numérique is able to control when and which sounds are triggered, but the audience can move around with their devices and decide whether to participate or not. The common denominator of these examples in literature and our own experience is that musicians are happy to let the audience control parts of their performance using TMAP. However, they do not want to grant them too much control and not on essential aspects of their music or pure artistic aspects.

From a creative perspective, designing a TMAP performance using smartphones, led to several artistic techniques that build on this particular technology: (1) using the display of the smartphone as a light show, (2) using randomness for visuals and sound samples in a musical composition, and (3) using unintended sound effects caused by the diverging latency and different lengths of sound samples played by different devices at the same time.

To some extent, randomness is always part of a TMAP performance. In Experience (Hodl et al., 2014, September 14–20), for instance, the randomness of the audience's participation behaviour was even simulated during the composition process to see how it might sound. In Poème Numérique, however, not the randomness of the audience but the technology was essential and inspiring for the artist. When she attended the development workshops and the public test, she saw and experienced the technology. That inspired her to use randomness caused by the technology (i.e. varying latency of different devices) and enabled by the technology (i.e. using random blinking colours on the smartphone screens).

We know that new digital musical instruments can creatively inspire composers (Hödl, 2019; Zappi and McPherson, 2014). In Poème Numérique, the smartphone was the predefined technology for audience participation and became the digital music instrument that creatively inspired the artist during composition. During the performance, all spectators' smartphones formed the extended digital music instrument of the artist, who controlled the distributed devices from the stage.

Concerning the creative practice of the artist, the iterative development and continuous testing of the technology was very important. The large public test proved to be essential and beneficial to the artist to better understand how to compose and how to use the Poème Numérique app. The rehearsals at the actual performance venue where important to develop the whole performance setting and optimise the smartphone app. The importance of an early and realistic rehearsing scenario became obvious when the artist decided to not use the pickup coils at all during the general rehearsal last minute. At this point, it was too late to change the concept or refine the technology. Thus, an essential part of the audience participation became Poème Numérique obsolete.

## 7. Conclusion

Our study identifies implications when using a smartphone for audience participation in live music. We used the smartphone as a central technology and design tool to develop a performance together with an artist. This included an ideation phase with potential concert-goers, iterative development and rehearsing throughout more than a year in workshops as well as a large-scale test.

At the end, we present Poème Numérique as smartphone app as well as a participatory performance concept including the preceding development process. We reflected on this process concerning technological issues, the experience during development and testing, and the involved artist's perspective. The artist was continuously learning about the technology to see what is possible, what happens when using it, and how it works. Moreover, she was inspired to try out new creative ideas during composition. After all, with the experience of Poème Numérique, the artist became curious and motivated to try out further performances and ideas using smartphone-based audience participation. On the critical side, there were certain points when the smartphone intervention did not match the artist's musical expectations. In general, she was cautious about influencing the quality of sound and music and she also rejected audience participation elements even at the final rehearsal.

The smartphone became the design tool, the technical device and the musical instrument at the same time. ?>Usually, non-related different activities and knowledge such as the creative practice and technical expertise became closely intertwined in the course of the performance development process. This resulted in the necessity of interplay between the acts of designing, developing, composing, and rehearsing. Thus, we value the potential smartphone technology can bring to a music performance. But we also highlight the importance of intense and iterative rehearsing with the technology and involve the artist as much as possible even during technical tests.

### 7.1. Limitations and future work

The workshops we used to develop the Poème Numérique app were highly influenced by the experts' and the artists interests. A different mixture of experts or a change of the performer may have come to a different result concerning the performance concept and the experience of the development process.

Due to time limit and availability, we ran our public test during the briefing of a lecture for computer science students in the second semester of their bachelor studies. Therefore, the limiting factors are that nearly all test participants were computer science students, most of them were male (79,5%), and most of them were between 18 and 25 years old (85,4%).

This study focuses on the development process and the evaluation with a large public test audience. In a future study, we will investigate the experience of an audience at the actual live performance to extend our results.

## Footnotes

1 https://youtu.be/6API0Xr2LV8 (last accessed 3 September 2019).

2 https://youtu.be/J-BfwfYdXG0 (last accessed 3 September 2019).

3 https://youtu.be/9SQtmqEXPKU (last accessed 3 September 2019).

4 https://youtu.be/DC6iOkqWbpg (last accessed 3 September 2019).

5 http://www.piglab.org/breakingthewall (last accessed 3 September 2019).

6 http://www.piglab.org/breakingthewall (last accessed 3 September 2019).

7 http://www.indigo-inc.at (last accessed 3 September 2019).

8 http://xylobands.com (last accessed 3 September 2019)

9 http://www.xamarin.com (last accessed 3 September 2019).

10 The interviews were conducted in German, translations of quotes in this section were made by the authors.
